# Supplementary material for: A fault-intrusion-tolerant system and deadline-aware algorithm for scheduling scientific workflow in the cloud
Source: PeerJ Comput Sci. 2021 Nov 2;7:e747. doi: 10.7717/peerj-cs.747 (PMC8576554; doi:10.7717/peerj-cs.747)
Supplement: Supplemental Information 6 [file peerj-cs-07-747-s006.zip › Myproject1.1/docs/org/cloudbus/cloudsim/core/predicates/Predicate.html]

Predicate


JavaScript is disabled on your browser.


- Overview
- Package
- Class
- Use
- Tree
- Deprecated
- Index
- Help

- Prev Class
- Next Class

- Frames
- No Frames

- All Classes

- Summary:
- Nested |
- Field |
- Constr |
- Method

- Detail:
- Field |
- Constr |
- Method


org.cloudbus.cloudsim.core.predicates

## Class Predicate

- java.lang.Object
- - org.cloudbus.cloudsim.core.predicates.Predicate

- Direct Known Subclasses:
  :   PredicateAny, PredicateFrom, PredicateNone, PredicateNotFrom, PredicateNotType, PredicateType

  ---

    

  ```
  public abstract class Predicate
  extends java.lang.Object
  ```

  Predicates are used to select events from the deferred queue. This class is abstract and must be
  extended when writing a new predicate. Some standard predicates are provided.  
  The idea of simulation predicates was copied from SimJava 2.

  Since:
  :   CloudSim Toolkit 1.0

  See Also:
  :   `PredicateType`,
      `PredicateFrom`,
      `PredicateAny`,
      `PredicateNone`,
      `Simulation`

- - ### Constructor Summary

    Constructors

    Constructor and Description

    `Predicate()`
  - ### Method Summary

    Methods

    | Modifier and Type | Method and Description |
    | --- | --- |
    | `abstract boolean` | `match(SimEvent event)` The match function which must be overridden when writing a new predicate. |

    - ### Methods inherited from class java.lang.Object

      `clone, equals, finalize, getClass, hashCode, notify, notifyAll, toString, wait, wait, wait`

- - ### Constructor Detail


    - #### Predicate

      ```
      public Predicate()
      ```
  - ### Method Detail


    - #### match

      ```
      public abstract boolean match(SimEvent event)
      ```

      The match function which must be overridden when writing a new predicate. The function is
      called with each event in the deferred queue as its parameter when a
      `Simulation.select()` call is made by the user.

      Parameters:
      :   `event` - The event to test for a match.

      Returns:
      :   The function should return `true` if the event matches and should be
          selected, or `false` if it does not match the predicate.


- Overview
- Package
- Class
- Use
- Tree
- Deprecated
- Index
- Help

- Prev Class
- Next Class

- Frames
- No Frames

- All Classes

- Summary:
- Nested |
- Field |
- Constr |
- Method

- Detail:
- Field |
- Constr |
- Method
